# Efficient Removal of Hexavalent Chromium from an Aquatic System Using Nanoscale Zero-Valent Iron Supported by Ramie Biochar

**DOI:** 10.3390/nano11102698

**Published:** 2021-10-13

**Authors:** Xiangpeng Tan, Muhammad Shaaban, Jianwei Yang, Yajun Cai, Buyun Wang, Qi-An Peng

**Affiliations:** 1 School of Environmental Engineering, Wuhan Textile University, Wuhan 430073, China; xiangpengtan@163.com (X.T.); yangjw19991204@163.com (J.Y.); juniacai@163.com (Y.C.); wbuyun@126.com (B.W.); 2 Key Laboratory of Mountain Surface Processes and Ecological Regulation, Institute of Mountain Hazards and Environment, Chinese Academy of Sciences, Chengdu 610041, China; shabanbzu@hotmail.com; 3 Department of Soil Science, FAS&T, Bahauddin Zakariya University, Multan 60800, Pakistan

**Keywords:** heavy metals, hexavalent chromium, nanoscale zero-valent iron, biochar, removal mechanism

## Abstract

In this study, ramie biochar (RBC) was used to activate nano zero-valent iron (nZVI) to enhance hexavalent chromium (Cr(VI)) removal. The best results were obtained at a pyrolysis temperature of 600 °C, a biochar particle size of < 150 μm, and an iron to carbon ratio = 1:1. Under the optimal conditions, the removal of Cr(VI) by RBC600-nZVI (98.69%) was much greater than that of RBC600 (12.42%) and nZVI (58.26%). Scanning electron microscopy (SEM), X-ray diffraction (XRD), Fourier transform infrared spectroscopy (FT-IR), and X-ray photoelectron spectroscopy (XPS) revealed that the reaction mechanism at the Fe and Cr interface was a multiple interaction mechanism with reduction dominated, adsorption, and co-precipitation simultaneously. The enhanced performance of RBC600-nZVI resulted from the effective dispersion of nZVI on the surface of RBC600, therefore increasing the adsorption activity sites. At the same time, RBC600 and nZVI exerted a synergistic influence on the composite structure, which jointly promoted the reduction reaction of Cr(VI) and removed more Cr(VI). This study shows that RBC-nZVI is a potentially valuable remediation material that not only provides a new idea for the utilization of ramie waste, but also effectively overcomes the limitations of nZVI, thus, achieving efficient and rapid remediation of Cr(VI).

## 1. Introduction

Environmental pollution of heavy metals is a devastating problem all over the world. Several industries release heavy metals into the environment. Chromium (Cr) is extensively used in industries for divergent purposes such as dye manufacturing, electroplating, paper production, leather tanning, and synthesis of paints [1,2]. Indecorous discarding and insufficient storage result in the entry of a huge amount of Cr into the environment and cause numerous chances of soil and water pollution in industrial areas [3,4]. Naturally, Cr in the environment is found in Cr(VI) and Cr(III) forms. The Cr(VI) is more concerned because of relatively more soluble, mobile, and toxic than Cr(III) [5,6]. In addition, Cr(VI) has the potential to cause health-related problems ranging from minor skin irritation to lungs cancer [7]. Thus, the elimination of Cr(VI) from water resources is vital and an urgent environmental concern.

Scientists have devised the regulations related to limitations of heavy metals released into the environment, but novel efficient techniques are required to be investigated for the Cr(VI) removal from the aqueous environment. Nanometer zero-valent iron (nZVI), based on its distinctive characteristics such as highly reactive, and large surface area, has been profoundly used for the elimination of Cr from aqueous systems. Nevertheless, the reactivity of nZVI decreases over time due to its agglomeration and oxidization [8,9]. Researchers have devised various techniques to avoid such processes of iron (Fe) nanoparticles. The most commonly used techniques are utilizing inactive metals, surfactants, inorganic clay minerals, organic biomass materials, etc. [9]. Unfortunately, the modification of some of the above-mentioned materials may cause secondary pollution or reduce the reaction efficiency. Therefore, it is particularly important to prepare stable, dispersed, and environment-friendly modified nZVI [10,11]. Recently, the environment-friendly material biochar (BC) as the carrier material of nZVI has attracted extensive attention [12], not only because of its wide source and low cost, but also because of its high specific surface area, rich functional groups, and pores [13], which can make nZVI form on the surface of biochar without agglomeration and oxidation [9]. It is a technology with an application with the huge prospect of Cr elimination from the polluted environment systems. Currently, there have been several studies on the feasibility of removing Cr(VI) from aquatic solutions using biochar loaded with nZVI. The results indicate that BC-nZVI can act as a cost-effective adsorbent to remove Cr(VI) from wastewater. Some of the most recent works on BC-nZVI composites are summarized in Table 1. However, there are few studies on the functional properties of BC-nZVI composites [14]. The removal efficiency of loaded nZVI is a function of the surface area of loaded materials, iron content and exposed functional groups [14]. Therefore, it is important to optimize the surface of BC to achieve the maximum removal. The effects of preparation temperature, particle size and iron loading for the removal efficiency of Cr(VI) through BC application need further investigations. The production of Ramie (*Boehmeria nivea*; China grass, fiber yielding plant) is huge in China, and the cellulose content of ramie is very rich [15,16]. The by-products of cellulose pyrolysis can remove heavy metal ions in wastewater. Therefore, whether from the economic or environmental aspects, it has important application value to make biocarbon from ramie straw waste. The overall goal of this study was to increase the adsorption performance of Cr(VI) by ramie biochar (RBC) combined with nZVI (RBC-nZVI composite). The removal efficiency of Cr(VI) by RBC-nZVI composite under different preparation conditions was compared through batch adsorption experiments in the laboratory. The mechanism of Cr(VI) removal by RBC-nZVI composite was revealed by characterization and analysis using various advanced techniques. The specific research contents are as follows: (1) the effects of pyrolysis temperature, particle size, and iron loading on the physicochemical properties of RBC-nZVI composite were determined, (2) the adsorption characteristics of RBC-nZVI composite and the influence of pH and other environmental factors on Cr(VI) removal were determined, and (3) the mechanism of Cr(VI) removal by RBC-nZVI composite was elucidated.

## 2. Materials and Methods

### 2.1. Chemicals

In order to perform experiments, we used the following reagents: ferrous sulfate heptahydrate (FeSO_4_·7H_2_O), anhydrous ethanol (C_2_H_6_O), hydrochloric acid (HCl), sodium hydroxide (NaOH), sodium borohydride (NaBH_4_), potassium dichromate (K_2_CrO_4_), acetone (C_3_H_6_O), diphenylcarbazide (C_13_H1_4_N_4_O), phosphoric acid (H_3_PO_4_), and sulfuric acid (H_2_SO_4_) were instead purchased from Shanghai Guoyao reagent group, Shanghai, China. Analytical grade chemicals were used in the experiments. Whenever needed, ultra-pure water was used in the experiment.

### 2.2. Preparation of Biochar

Biochar was prepared by slow pyrolysis of biomass under closed anoxic conditions. The peeled waste ramie stalk was used as biomass raw material. Before pyrolysis, the biomass was washed with distilled water, and subsequently dried in an oven (GZX-9030MBE, Shanghai Bosun Industrial Co., Ltd., Heifei, China) at 60 °C for 72 h, and then milled into a powder (<300 µm) in a grinding chamber (RS-FS1811, Hefei Royalstar Small Appliance Co., Ltd., Heifei, China). The crushed ramie straw was oven-dried at 60 ℃ for 12 h in a ceramic crucible. The biomass was placed in the vacuum tube furnace (1500X-G.S.L., Hefei Kejing Material Tech. Co., Ltd., Heifei, China) and pyrolyzed at 300, 400, 500, 600, and 700 °C under anoxic conditions for 2 h. After that, raw biochar was treated with 1 mol/L HCl solution for 12 h and the effluent was washed with distilled water until the pH reached neutral. The HCl-treated biochar was oven-dried at 60 °C for 12 h and, in order to prevent moisture contact, stored in a desiccator. The granules of biochar were crumbled and ground to make a powder using a mortar and pestle, and sieved for obtaining the following particle sizes: 300–600 µm, 150–300 µm, 75–150 µm, and <75 µm.

### 2.3. Preparation of nZVI

The nanoscale zero-valent iron (nZVI) was prepared using the method of liquid-phase reduction [27]. Simply, an aqueous solution of NaBH_4_ was added into FeSO_4_·7H_2_O solution. The ferric ion was reduced resulting an nZVI as shown in the Equation (1):

(1) $$Fe^{2+}+2BH_{4}^{-}+6H_{2}O\to Fe^{0}+2B{\left(OH\right)}_{3}+7H_{2}↑$$

Briefly, we dissolved FeSO_4_·7H_2_O (0.05 M) in the ethanol and anaerobic water solution (solution of anaerobic ethanol and anaerobic water was prepared with 1:4, *v*/*v* ratio) contained in a three-neck flask. After that, stirring was performed with ambient N_2_ gas for 30 min. The NaBH_4_ (0.2 M) was dissolved in ultrapure water using a separating funnel, and gently poured in the FeSO_4_·7H_2_O solution. Afterwards, vigorous stirring of the mixture was performed for 1 h under anaerobic conditions (N_2_ gas was used to develop anaerobic conditions) during the entire procedure to avoid the oxidation process of nanoparticles. Following the completion of the reaction, the precipitates of nZVI were rinsed using anoxic ultrapure water and anhydrous ethanol three times for the removal of excessive NaBH_4_. The nZVI precipitates were dried following the method of vacuum-drying-chamber for 12 h and subsequently kept in a vacuum drying container.

### 2.4. Preparation of RBC-nZVI

**Figure 1:**
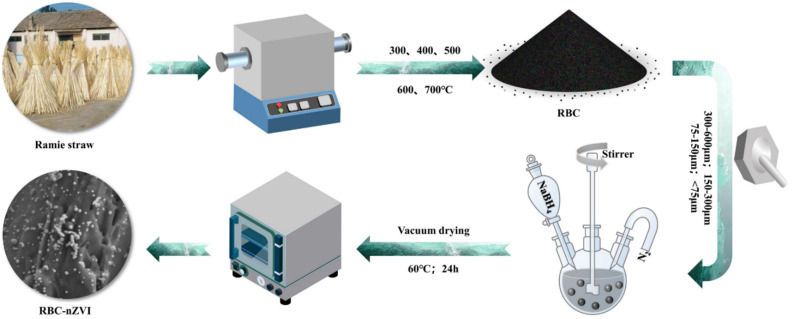
Preparation process of RBC/nZVI composite.

RBC-nZVI was synthesized according to the method described by Su et al. [28] with some modifications. Initially, ferrous sulfate (FeSO_4_·7H_2_O) and biochar were pooled in 75 mL of anaerobic water (adjusted pH of the solution was 4.0) and agitated on an electric shaker at 150 rpm for 24 h at 25 °C. After that, the solution was moved into a three-neck round bottom flask which already had a volume of 25 mL of ethanol. The N_2_ gas was purged into the solution, while concurrently, a robust stirring was performed for 30 min to remove dissolved O_2_. Then, NaBH_4_ solution was mixed dropwise into the slurry under strong stirring conditions. The fabrication process is shown in Figure 1. The prepared products were used for subsequent characterization and experiments.

### 2.5. Characterization

The characterization of material was carried out by adopting several techniques including Scanning Electron Microscopy (SEM, Zeiss Sigm 300, Los Angeles, CA, USA), Fourier Transform Infrared (FTIR, 6700, Therm. Scient. Nicolt, Steinheim, Germany), X-ray powder diffraction (XRD, D8 Brukr Adv., Oberkochen, Germany), and X-ray photoelectron spectroscopy (XPS, Therm. Scient. K-Alpha, Boston, MA, USA). The dispersion, surface morphology, microstructure of nanoparticles were tested by SEM. To analyze the FTIR spectra, NZVI and biochar were mixed and powder of potassium bromide (KBr) was added with a mass ratio of 1/100. A wave number ranging from 400 to 4000 cm^−1^ was noted at a resolution of 4 cm^−1^ and 64 inter-ferogram. The XRD spectra were obtained to analyze the crystal structure of nZVI and biochar. The XRD analysis had the capability to identify at a scanning speed of 5 min^−1^ ranging from 5 to 80. The identification of chemical composition and chemical valent changes before and after Cr(VI) remediation was validated on X-ray photoelectron spectroscopy.

### 2.6. Batch Experiments

#### 2.6.1. Influence of Preparation Conditions

For the comparison of the effects of different RBC and RBC-nZVI on Cr(VI) elimination from aqueous solution, the reaction for the removal of Cr(VI) was conducted in a series of conical flasks. The reaction system was sampled and analyzed at 0, 5, 10, 20, 30, 40, 50, and 60 min. The content of Cr(VI)_e_ was analyzed, and the Cr(VI) elimination was was calculated using Equation (2):

Cr(VI) removal = Cr(VI)_0_ − Cr(VI)_e_/Cr(VI)_0_ (2)

A volume of 100 mL of 20 mg/L Cr(VI) solution was taken into each conical flask (250 mL), and then 1 g/L of RBC-nZVI particles was added into each conical flask. The reaction was carried out on a constant temperature oscillator with a rotating speed of 150 r/min at 25 °C. The samples were taken at regular intervals and separated through a polytetrafluoroethylene membrane (0.22 µm). The Cr(VI) concentration was analyzed by diphenyl carbazide spectrophotometry [29].

#### 2.6.2. Influence of Environmental Factors

To explore the effects of different reaction conditions on the removal of Cr(VI) from aqueous solution, for example, initial Cr(VI) concentration (mg/L), material dosage (g/L), reaction temperature (°C), and initial pH of the solution, the optimal conditions for the reaction were determined. In the batch experiment, the dosage of the composite was set as 0.5, 0.8, 1.0, 1.3, and 1.5 g/L, and the initial concentration of Cr(VI) was 20 ± 1 mg/L; other reaction conditions remained unchanged. In the batch experiment, the initial concentration of Cr(VI) was set to 10 ± 1, 20 ± 1, 40 ± 1, 60 ± 1, 80 ± 1 mg/L, whereas the dosage of the composite was 1.0 g/L, and other reaction conditions remained unchanged. In the batch experiment to explore the effect of reaction temperature, the reaction temperature was selected as 25, 35, 45, 55 °C, the dosage of composite was 1.0 g/L, and other reaction conditions remained unchanged. In order to explore the effect of the initial pH of the solution, the initial pH of the solution was set as 3, 5, 6, 7, and 9, the dosage of composite material was 1.0 g/L, and the original concentration of Cr(VI) was 20 ± 1 mg/L, other reaction conditions remained unchanged. Two parallel control groups were set for the above reactions to explain the error range in the reaction process.

## 3. Results and Discussion

### 3.1. The Characterization of Biochar and Biochar-Supported nZVI

#### 3.1.1. SEM

**Figure 2:**
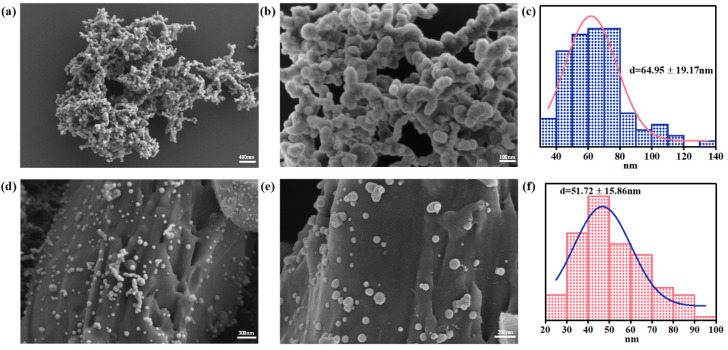
SEM images and corresponding particle size distribution of nZVI (**a**–**c**) and RBC600-nZVI (**d**–**f**). The insertion: the nanoparticle size distribution, calculated with Nano Measurer and 100 particles were selected for each sample.

The morphology and structure of the nZVI and RBC600-nZVI were identified through SEM analysis (Figure 2). The particles of nZVI produced by liquid-phase reduction had the size of nanometer scale, were spherical in shape, forming protruding chain-like aggregates (Figure 2a,b), which was primarily because of the interaction of the surface effects and the static magnetic forces. The analysis of the nano-measurer displayed that the average size of nZVI was 64.95 ± 19.17 nm. We confer that enhanced dispersion and smaller size of nZVI particles resulted due to the amalgamation of RBC (Figure 2c,d). The mean particle diameters were 51.72 ± 15.86 nm for RBC600-nZVI. Furthermore, there was not any prominent aggregation on RBC600-nZVI. The results show that the particles of nZVI were markedly immobilized on the surface of RBC.

#### 3.1.2. FTIR

**Figure 3:**
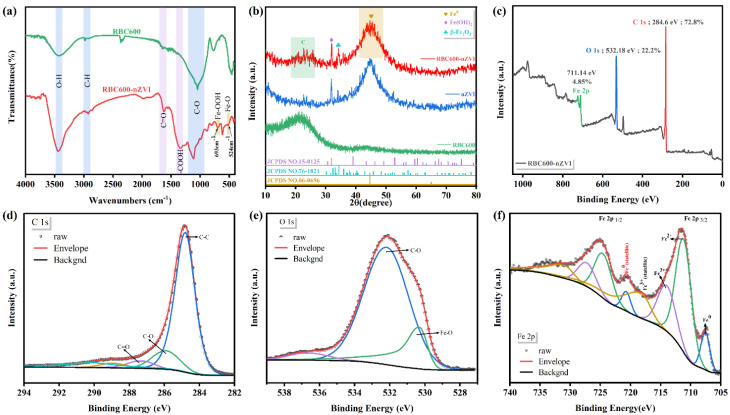
FTIR spectra pertaining to the RBC600 and RBC600–nZVI (**a**); XRD patterns of RBC600, nZVI, and RBC600–nZVI (**b**); XPS spectra of RBC600–nZVI prior to reaction with Cr(VI): Full survey (**c**), C 1s (**d**), O 1s (**e**), and Fe 2p (**f**) binding state levels.

The functional groups in RBC600 and RBC600-nZVI were determined by implementing an FT-IR technique (Figure 3a). An absorption peak obviously occurred at 3440 cm^−1^, which ranged between 3200 and 3500 cm^−1^, representing the intermolecular stretching of the hydrogen (H) bonding and thereby representing the peak of the hydroxyl group [30]. Markedly, the hydroxyl groups of RBC600-nZVI were ominously augmented as compared with the RBC600, which supported relatively more adsorption pockets for Cr(VI) adsorption [31]. There was a sharp peak at 2924 cm^−1^ which contributed to C-H stretching vibration (–CH_3_ or –CH_2_ group) [30,32]. The band around 1115 cm^−1^ was relevant to the group pertaining to the C–O stretching vibrations [3]. The FT-IR spectrum pertinent to RBC600-nZVI presented the typical peaks of iron (Fe) species, comprising the bands of Fe-O stretching vibration at ~524 cm^−1^ [1]. In addition, the peak of 693 cm^−1^ displayed the occurrence of Fe-OOH [4]. In comparison to RBC600, the RBC600-nZVI reflected two additional absorption peaks at 1341 and 1625 cm^−1^, indicating -COOH and C=O, respectively [5,33].

#### 3.1.3. XRD

The RBC600, nZVI, and RBC600-nZVI particles were characterized by XRD (Figure 3b). The RBC600 displayed a diffraction peak at 22.0° [4]. The sample of nZVI exhibited a principal peak at 44.7° attributing to (110) planes of a-Fe^0^ (JCPDS Number: 06-0696) [7], the peaks at 31.9° and 34.4° pertained to the planes of (113) of Fe(OH)_2_ (JCPDS Number: 15-0125) and (113) of β-Fe_2_O_3_ (JCPDS Number: 76-1821), respectively, which is a characteristic core-shell structure of nZVI [34]. For the RBC600-nZVI, the peaks at 22°, 31.9°, 34.4°, and 44.7° were observed, displaying an effective production of nZVI on the surface of RBC600 [19].

#### 3.1.4. XPS

The electronic valence of the elements in the RBC600-nZVI were examined through XPS [1]. Full survey spectrum of RBC600-nZVI showed the peaks at 284.8 eV pertaining to C, 532.2 eV related to O, and 711.1 eV pertinent to Fe elements (Figure 3c). The ratio of C, O, and Fe elements to total surface iron atoms was about 72.8, 22.2, and 4.85%, respectively. Figure 3d shows peaks at 284.8, 285.9, 287.3, 288.9 eV, and 290.2 eV, corresponding to C–C, C–O, C=O, and O-C=O, and C–COOR, respectively. After the deconvolution of C 1s, C–C, C–O, C=O, O-C=O, and C–COOR were found to account for 71.54, 12.59, 8.09, 4.92, and 2.85%, respectively. Figure 3e represents the O 1s spectra of XPS, which comprised peaks at 530.3 and 532.2 eV displaying Fe-O and C–O, respectively. After the deconvolution of O 1s, Fe-O and C–O were found to account for 77.91 and 17.87%, respectively. The XPS analysis confirmed that Fe supported on RBC600-nZVI exists in different oxidation states [21]. The Fe 2p spectrum of the material revealed having eight divergent peaks (Figure 3f). The peaks having binding energies of 727.2 eV, 724.5 eV, and 720.69 eV reflected the Fe 2p1/2, while the peaks having binding energies of 713.97 eV, 711.14 eV, and 707.55 eV were pertinent to the Fe 2p3/2. The binding energy of the Fe 2p1/2 and Fe 2p3/2 at 727.23 eV and 713.97 eV corresponded to trivalent Fe. The binding energy of the Fe 2p1/2 and Fe 2p3/2 at 724.52 eV and 711.14 eV corresponded to divalent Fe. The peak at 707.6 eV was related to Fe^0^, indicating that the existence of Fe^0^ species on the RBC600-nZVI surface. The peak area ratio of Fe^0^: Fe(II): Fe(III) was 1: 2.4: 2.5. This denotes that the nZVI surface was certainly wrapped with an oxide layer having a thickness of <10 nm [35,36]. Nevertheless, earlier studies illustrated that the surface oxide layer possesses several active adsorption sites [37], which is supportive of the adsorption of Cr(VI) [38].

### 3.2. Removal of Cr(VI) by RBC

**Figure 4:**
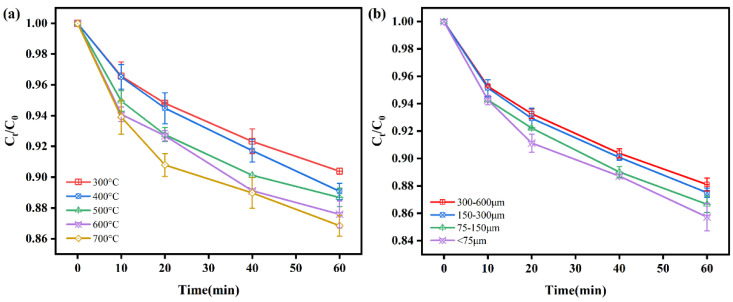
Cr(VI) removal by RBC prepared at different pyrolysis temperatures (**a**); RBC with different particle sizes (**b**); experimental conditions: initial Cr(VI) concentration 20 mg/L, RBC dosage 0.5 g/L, T = 25 °C, pH = 5.

The removal performance of RBC at different pyrolysis temperatures is shown in Figure 4a. The treatment effect of RBC had no significant (*p* < 0.05) difference pyrolyzed at 400, 500, 600, and 700 °C. The treatment effect of RBC600 and RBC700 was only 2.54 ~ 3.28% higher than RBC300. At 60 min, Cr(VI) removal rates by RBC pyrolyzed at 300–700 °C were 9.88, 10.92, 11.32, 12.42, and 13.16%, respectively. The RBC600 was selected to study the impact of particle size on Cr(VI) elimination by RBC (Figure 4b). During the whole reaction period, there were no significant (*p* < 0.05) differences for the removal efficiency of Cr(VI) by biochar with different particle sizes. In consistence with the literature, the above results displayed that Cr(VI) elimination by RBC was insignificant.

### 3.3. Effects of Preparation Conditions on Cr(VI) Removal

**Figure 5:**
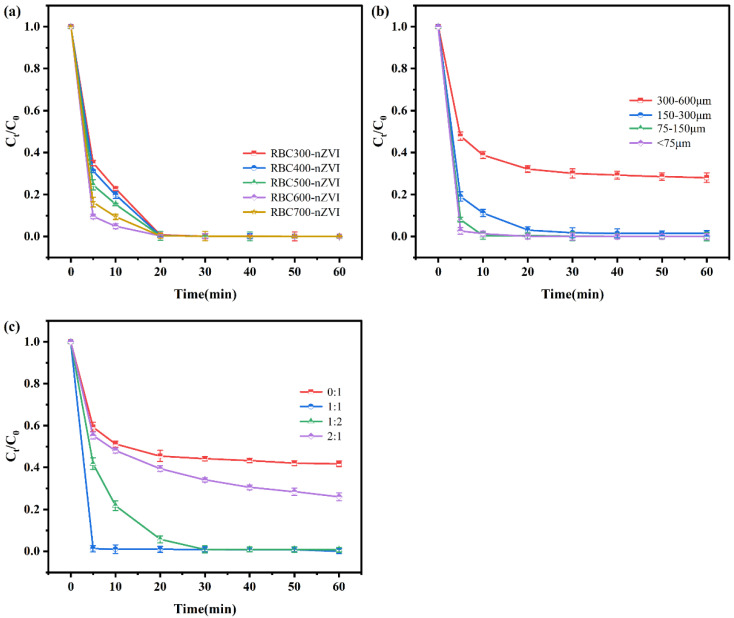
The removal of Cr(IV) by nZVI loaded with RBC600 prepared at different pyrolysis temperatures (**a**); NZVI loaded with different particle size RBC600 (**b**); different mass ratios of RBC600 to nZVI (**c**). Vertical bars denote the standard deviations of the means (*n* = 2). Experimental conditions: initial Cr(IV) concentration 20 mg/L, RBC-nZVI dosage 1 g/L, T = 25 °C, pH = 5.

Biochar produced at different pyrolysis temperatures (RBC300-RBC700) were utilized to support nZVI particles for Cr(VI) removal. The RBC300-nZVI, RBC400-nZVI, RBC500-nZVI, RBC600-nZVI, and RBC700-nZVI displayed great efficacy for Cr(VI) removal (Figure 5a). The Cr(VI) removal was quick during the first 5 min, whereas the Cr(VI) removal performance of RBC300-nZVI~RBC700-nZVI after 20 min was almost the same (around 100%). The fastest removal efficacy of 90.44% was attained with RBC600-nZVI at 5 min, which was significantly higher than other treatments (*p* < 0.05). Moreover, Table 1 shows the removal ability of other biochar-modified nano-zero-valent iron materials for Cr(VI). Compared with them, the removal time of RBC600-nZVI is short and the removal efficiency is high. Therefore, RBC600 was selected to explore the removal effect of Cr(VI) by nZVI supported by RBC with different particle sizes. Figure 5b shows the performance of the four types of modified biochars (300–600 μm, 150–300 μm, 75–150 μm, and <75 μm) for the removal of Cr(VI). The results displayed that the effect of nZVI treatment with RBCs of 300–600 microns (71.96% at 60 min) was significantly lower than that of the other three treatments (all above 98%) (*p* < 0.05), among which RBCs with a particle size of less than 75 microns loaded nZVI works best, reaching the peak first. This may be because the smaller biochar particles (<150 µm) prevented the aggregation and oxidation of iron on the surface, and therefore the increased particle size led to the passivation of the composite due to aggregation and oxidation of iron particles.

The RBC600 with a particle size of <150 microns was selected to study the influence of the mass ratio of RBC600/nZVI on the Cr(VI) elimination by RBC600-nZVI. The divergent mass ratios of RBC600/nZVI (0:1, 1:1, 1:2, and 2:1 ratio) highly influenced the removal of Cr(VI) by RBC600-nZVI (Figure 5c). The results illustrated that the efficacy of RBC600-nZVI (1:1) (100%) for the alleviation of Cr(VI) was approximately 41.74% higher than that of bare nZVI (RBC600:nZVI = 0:1) (58.26%) and 26.08% higher than that of the RBC600-nZVI (2:1) (73.92%). Noticeably, nearly 100% of Cr(VI) alleviation was achieved by RBC600-nZVI (1:1) during the first 5 min. Moreover, it is noteworthy herein that both the reaction rate and elimination efficiency of RBC600-nZVI (1:1) for Cr(VI) were greater than the other mass ratio complexes with the equal mass of nZVI (500 mg/L). These results show that nZVI presence on the surface of biochar certainly plays a pivotal role in the alleviation of Cr(VI) [39]. When using nZVI alone, severe agglomeration (Figure 2a,b) hindered the reaction, resulting in the decreased reducing potential of the iron particles. When the content of RBC600 was high, BC blocked the active site of nZVI. When the mass ratio of BC/Fe was 1:1, nZVI particles were uniformly dispersed on the surface of RBC600, which had the highest reduction/charge transfer reactivity. Therefore, the subsequent experiment used mass ratios of RBC600 to nZVI = 1:1.

### 3.4. Effects of Physicochemical Factors on Cr(VI) Removal

**Figure 6:**
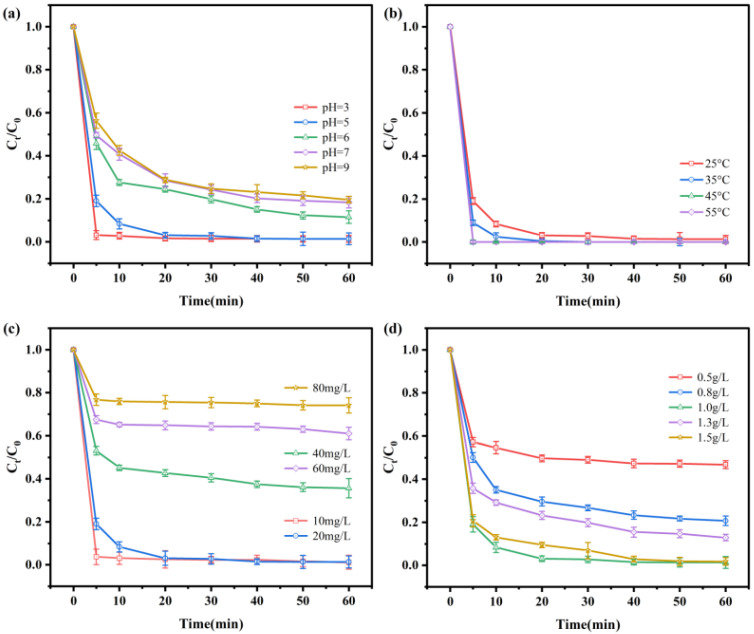
Cr(VI) removal at various pH values (**a**); at various temperature (**b**); at various initial concentration (**c**); at various dosages (**d**). Experimental conditions: initial Cr (IV) concentration 20 mg/L, RBC-nZVI dosage 1 g/L, T = 25 °C, pH = 5. Vertical bars denote the standard deviations of the means (*n* = 2).

The RBC600-nZVI displayed prominent results for pH-dependent removal of Cr(VI) (Figure 6a). The Cr(VI) removal dramatically decreased (*p* < 0.05) with the increment of initial pH (3, 5, 6, 7, and 9). This result is in accordance with the earlier studies [26,27,40]. The results indicated that a lower pH is more appropriate for the alleviation of Cr(VI) through implementing RBC600-nZVI. When the pH was 3, the removal rate was close to 100% at 5 min. Studies have shown that pH impacts the Cr(VI) existence [41]. The predominant forms of Cr(VI) were HCrO_4_^−^, Cr_2_O_7_^2−^, and CrO_4_^2−^ between pH 2.0 and 8.0; the acidic environment facilitates the removal [42]. In addition, with respect to pH, nZVI is more likely to release reducing electrons under acidic conditions, supporting Cr(VI) elimination and conversion to a lower valent state [43]. Increasing pH further enhanced Fe(III)/Cr(III) hydroxide co-precipitating onto nZVI reactive sites, hence decreasing Cr(VI) removal. The hydroxides and oxide layers are readily developed on the surfaces of iron nanoparticles under alkaline conditions, which impedes the Cr(VI) elimination through iron nanoparticles [44]. It is different from the literature that the treatment effect was still 80% at pH 9 in the present study. Since the difference between the treatment’s effect at pH 5 and 3 was not significant (*p* > 0.05), a pH of 5 was selected for subsequent experiments.

Temperature is also an imperative factor for the reaction of systems. The production of RBC600-nZVI was intended to react with a volume of 50 mL of 20 mg/L aqueous Cr(VI) solution at divergent gradients of temperatures (i.e., 25, 35, 45, and 55 °C), which demonstrated varied removal rates (Figure 6b). The removal efficiency increased continuously with the increase of temperature raised from 25 to 55 °C. The removal rate of 45 (100%) and 55 (100%) at 5 min was significantly (*p* < 0.05) higher than that of 25 (80.96%) and 35 (91.05%). This was possibly due to higher temperature conducive to enhance the diffusion Cr(VI) ions, thus improving the contact possibility of Cr(VI) ions to the adsorption pockets [45]. In addition, the reduction reaction of Cr(VI) by nZVI is an endothermic process [46], and the increase in temperature enables the electron transfer from nZVI to Cr(VI) [47].

The initial concentration of metals also has a great influence on their removal through treatments from the system. In the present study, we investigated the effects of initial Cr(VI) concentrations ranging from 10 to 80 mg/L, on its alleviation from aqueous solution. The initial Cr(VI) concentrations of 10 and 20 mg/L were completely removed within 60 min (Figure 6c). The removal efficiency for Cr(VI) decreased with the increased initial concentration of Cr(VI). This was primarily due to the limitation of active sites of a certain amount of nZVI particles. With the increase of the initial concentration of Cr(VI), the available active sites are also occupied. Moreover, the reaction of Cr(VI) with nZVI became more intensive as the concentration increased, leading to the fast formation of Cr–Fe inactivating layer, thus enclosing the nZVI and blocking the electron transfer. The compound/hydroxide passivation layer inhibited the reaction.

Application rate (dose) of RBC600-nZVI markedly influenced the removal of Cr(VI) from the aqueous solution. The effect of RBC600-nZVI dosage on its capability for the Cr(VI) removal was examined in the range of 0.5–1.5 g/L. Results demonstrated that the removal efficiency of 1 g/L and 1.5 g/L was significantly (*p* < 0.05) higher than 0.5 g/L, 0.8 g/L, and 1.3 g/L (Figure 6d). It can be stated that as the application dose increased, the removal capacity of RBC600-nZVI for Cr(VI) alleviation increased obviously, except for 1.3 g/L. This was mainly due to the increased application dose of RBC600-nZVI that increased the total available active sites for Cr(VI). Taken together, 1 g/L was the best choice for the removal of Cr(VI) in the present study.

### 3.5. Removal Mechanism

The RBC600-nZVI substantially removed Cr(VI) from the aqueous solution. Several factors, processes, and mechanisms attributed to the removal of Cr(VI), mainly galvanic interactions, electrostatic attraction, and precipitation redox [5]. To probe and validate the removal mechanisms of Cr(VI), both XRD and XPS were used in order to examine the reaction products.

**Figure 7:**
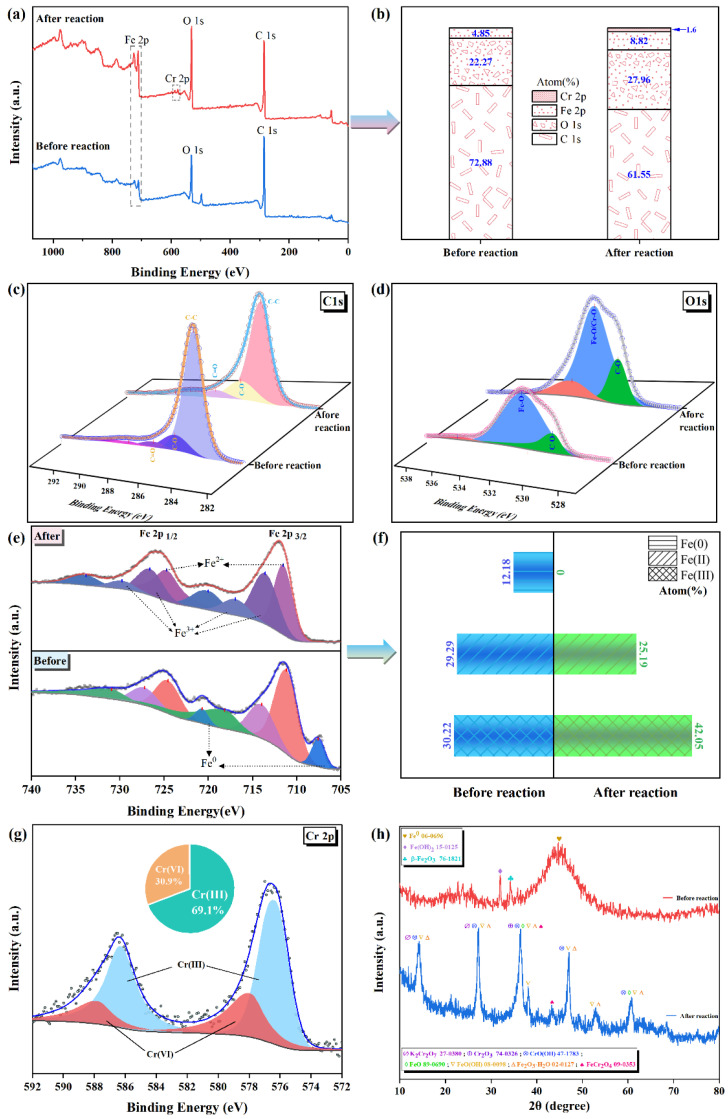
XPS spectra of RBC600-nZVI prior and post reactions with Cr(VI). (**a**) The full survey, (**b**) Content change of the surface atomic ratio of RBC600-nZVI before and after reaction with Cr(VI); (**c**) C 1s (**d**) O 1s and (**e**) Fe 2p binding state gradients; (**f**) The change of the specific content of iron in various valence states before and after the reaction of RBC600-nZVI with Cr(VI); (**g**) XPS spectra of Cr 2p after reaction with Cr(VI); (**h**) The XRD analysis of RBC600-nZVI before and after reaction with Cr(VI).

To understand the removal mechanism of Cr(VI), the XPS was employed to investigate the chemical transformations of RBC600-nZVI prior and post-reaction. The full scan of the XPS spectra illustrated obvious results for the RBC600-nZVI before and after the reactions and changes in the atomic ratio of the elements on their surface (Figure 7a,b). After the reaction with Cr(VI), the proportions of iron and oxygen increased by 5.69 and 3.97%, respectively. Reduction of carbon content indicates that RBC600 participates in the removal of Cr(VI) to a certain extent, which may be reduction or complexation. The research has demonstrated that the functional groups, for instance, phenolic and -COOH groups, and C matrix of biochar participated in Cr(VI) reduction [48]. The changes in the content of C–C, C–O, C=O, O−C=O, and C–COOR (Figure 7c,d) further indicates that a complex reaction may have occurred. The increase in the atomic ratio of iron indicates that the zero-valent iron nucleus is involved in the reaction. After the reaction, the Fe^0^ peaks disappeared (Figure 7e,f), suggesting that nZVI participated in the reaction and was oxidized by Cr(VI). Additionally, Fe(II) content decreased by 4.1%, while Fe(III) contents increased by 11.83%, proving that Fe(II) contributed to the reaction and oxidized to produce Fe(III). A new peak appeared at a binding energy of 580 eV, conjecturing that Cr uptake was carried out on the RBC600-nZVI surface [49]. The peaks of 576.6 and 587.2 eV matched to Cr(III), while the peaks of 578.0 and 587.9 eV were related to Cr(VI) (Figure 7g). The area ratios of Cr(III) and Cr(VI) were 69.1 and 30.9%, respectively, representing that both reduction and adsorption carried out during the reaction, however, the reduction process was dominant. The peaks at 577.0 and 589.0 eV showed that Cr(III) existed in the form of (Fe_x_Cr_1−x_) (OH)_3_ or Fe (OH)_3_-Cr (OH)_3_ [50].

To further investigate the removal mechanism of Cr(VI), the XRD technique was employed to identify the chemical transformations of RBC600-nZVI. The distinctive changes in RBC600-nZVI were observed post-reaction with Cr(VI) (Figure 7h). The XRD analysis for the RBC600-nZVI prior to the reaction with Cr(VI) displayed a distinct characteristic peak of Fe^0^, which disappeared dramatically after the reaction. The XRD analysis for the RBC600-nZVI after reaction showed the existence of K_2_CrO_4_, Cr_2_O_3_, CrO(OH), and FeCr_2_O_4_, which were not distinguished in the sample prior to the reaction occurring. The presence of Cr(III), Fe(III), and Fe(II) in RBC600-nZVI after reaction validated the happening of redox reaction between Cr(VI) ions and nZVI particles. Hence, it can be established that the reduction of Cr(VI) to Cr(III) was carried out in the form of Cr_2_O_3_, CrO(OH), and FeCr_2_O_4_ (Figure 7h). Moreover, the oxidation reaction changed the nZVI to FeO, FeO(OH), and Fe_2_O_3_. It is plausible that the results obtained through various techniques employed to characterize RBC600-nZVI evidently revealed that RBC600-nZVI was involved in the removal and reduction of Cr(VI) [23].

**Figure 8:**
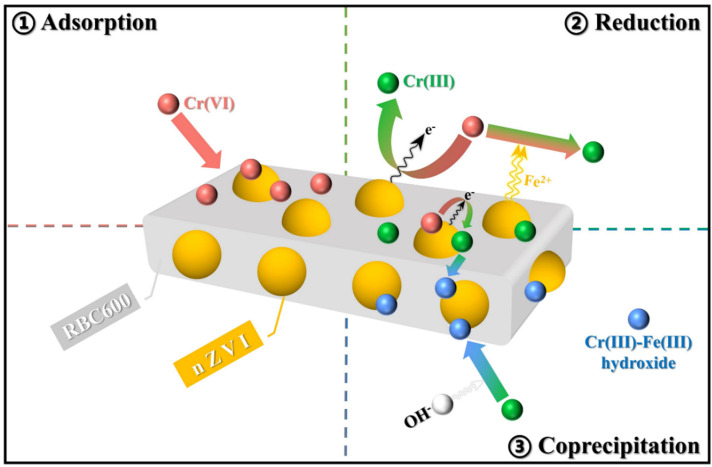
Proposed adsorption mechanisms of Cr(VI) by RBC600-nZVI.

The functional analysis of RBC600-nZVI composite revealed the potential mechanisms for Cr(VI) elimination from aqueous solution. The adsorption, reduction, and sedimentation took place simultaneously and cooperated with each other for the removal of Cr(VI) (Figure 8). The main reactions attributed in the process are as follows: (1) Adsorption: The adsorption of Cr(VI) oxyanions onto biochar carrier and onto oxide layers existent on the surface of nZVI. (2) Reduction: The majority of the adsorbed Cr(VI) species was simultaneously reduced to Cr(III) by Fe^0^ and Fe(II)-bearing secondary minerals coated on Fe^0^. In addition, Fe(II) dissolved by Fe^0^ oxidation (Fe^0^ corrosion) could still react with Cr(VI). (3) Co-precipitation: As the nZVI was oxidized, the pH of the solution progressively increased and OH^−^ aggregated, and Cr(III) combined with a large amount of OH^−^ induced during the reaction to form alloy-like Cr(III)-Fe(III) hydroxide and fixed in nZVI. Consequently, in the present scenario of the reaction system, Cr(VI) can be efficiently eliminated due to the synergistic effects of RBC600 and nZVI.

## 4. Conclusions

In the present study, we successfully prepared a nano-zero-valent iron (nZVI) supported by ramie biochar (RBC600-nZVI). The RBC600-nZVI displayed excellent Cr(VI) elimination efficiency from the aqueous solution. The nZVI was evenly dispersed and had a smaller particle size. The nZVI supported by RBC significantly improved the reaction efficiency and removal capacity for Cr(VI). The XPS and XRD analysis proved that the removal mechanism was mainly chemical reduction, with synergistic adsorption and complexation. Further research is suggested for using various types of biochar produced at different temperatures.

## Figures and Tables

**Table 1:** Removal of Cr(VI) from aqueous solutions by BC-nZVI composites.

Biomass	Conditions	Removal Performance	Ref.
Sewage sludge and sunflower seed shells	pH = 3; Cr(VI) = 50 mg L^−1^; Dosage = 1.0 g L^−1^; Reaction time: 90 min	95.00%	[17]
Trametes suaveolens	pH = 2; Cr(VI) = 50 mg L^−1^; Dosage = 5.0 g L^−1^; Reaction time: 90 min	100%	[18]
Oak wood	pH = 2; Cr(VI) = 50 mg L^−1^; Dosage = 0.04 g; Reaction time: 12 h	99.9%	[19]
Almond shell	pH = 2; Cr(VI) = 10 mg L^−1^; Dosage = 0.08 g; Reaction time: 60 min	99.8%	[20]
Flax straw	pH = 3; Cr(VI) = 100 mg L^−1^; Dosage = 0.05 g; Reaction time: 24 h	186.99 mg/g	[21]
Woody biomass of Prosopis juliflora	pH neutral; Cr(VI) = 10 mg L^−1^; Dosage = 0.02 g; Reaction time: 18 h	16.30 mg/g	[22]
Sewage sludge	pH = 4; Cr(VI) = 50 mg L^−1^; Dosage = 0.05 g; Reaction time: 24 h	31.53 mg/g	[23]
Sewage sludge and the starch	pH = 4; Cr(VI) = 30 mg L^−1^; Dosage = 1.5 g L^−1^;	98.8%	[24]
Rice straw	pH = 4; Cr(VI) = 20 mg L^−1^; Dosage = 0.05 g; Reaction time: 24 h	40.0 mg/g;	[25]
Cornstalk	pH = 5; Cr(VI) = 10 mg L^−1^; Dosage = 0.2 g L^−1^; Reaction time: 4 h	17.8 mg/g	[6]
Herb-residue	pH = 2; Cr(VI) = 20 mg L^−1^; Dosage = 0.2 g L^−1^; Reaction time: 4 h	98.71%	[26]

## Data Availability

The data presented in this study are available on request from the corresponding author.
